# Numerical Simulation on Seismic Behavior of Steel Fiber Reinforced Concrete Beam—Column Joints

**DOI:** 10.3390/ma14174883

**Published:** 2021-08-27

**Authors:** Ke Shi, Junpeng Zhu, Pengfei Li, Mengyue Zhang, Ru Xue, Tao Zhang

**Affiliations:** 1 School of Civil Engineering and Architecture, Zhengzhou University of Aeronautics, Zhengzhou 450046, China; shike@zua.edu.cn (K.S.); zhujunpeng@zua.edu.cn (J.Z.); lipf2021@163.com (P.L.); zhangmengyue@zua.edu.cn (M.Z.); xueru6239@163.com (R.X.); 2 Huadian Zhengzhou Mechanical Design Institute Co., Ltd., Zhengzhou 450046, China

**Keywords:** beam–column joint, steel fiber reinforced concrete, Opensees, seismic behavior

## Abstract

Steel fiber reinforced concrete (SFRC) is a novel material of concrete, which has a great potential to be used in practical engineering. Based on the finite element software Opensees, the main objective of this paper presented a numerical simulation method on investigating the seismic behavior of SFRC–beam-column joints (BCJs) through modifying the calculation method of joint shear and longitudinal reinforcement slip deformations. The feasibility and accuracy of the numerical modeling method were verified by comparing the computed results with experimental data in terms of the hysteresis curves, skeleton curves, feature points, energy dissipation, and stiffness degradation. And then, the influences of some key parameters on the seismic behavior of BCJs were investigated and discussed in detail. The parametric studies clearly illustrated that both adding the steel fiber and increasing the stirrup amount of joint core area could significantly improve the seismic behavior of BCJs. The axial compression ratio had limited influence on the seismic behavior of BCJs. Finally, based on the main factors (steel fiber volume ratio, stirrup amount, and axial compression ratio), a formula for predicting ultimate shear capacity is derived.

## 1. Introduction

In recent decades, the construction industry has developed rapidly, and a large number of buildings have adopted reinforced concrete frame structures. As the connecting member of beam and column, the joint part of the frame structure is the key part of force transmission and the weakest part under earthquake [1,2,3,4]. Under high shear stress, the joint zone will produce certain inelastic deformation after the beam longitudinal reinforcement yielding, mainly including shear deformation in the joint core zone and bond-slip deformation of the longitudinal reinforcement. The traditional method of simulating inelastic deformation was to apply an inelastic rotating spring to the end of the rod. Yang et al. [5] considered the bond-slip deformation of longitudinal bars by adding inelastic rotating springs to the ends of beams. Alath et al. [6] added zero-length rotating springs to the joint area to consider the influence of shear deformation of joints. Ashraf et al. [7] revised the model of Alath et al. [6] and adopted two kinds of inelastic rotating springs in the joint area to consider the influence of shear deformation of joints and bond-slip of longitudinal bars, respectively. However, the factors considered in the model are not comprehensive, which cannot accurately simulate the real stress state of the joint core area. To refine the joint finite element model, Fleury et al. [8] used the multi-component model to simulate the joint element, in which two parallel four-node elements were used to simulate the concrete and stirrup in the core area of the joint, respectively. The slender quadrilateral grid was used to simulate the bond-slip of longitudinal bars in joints. Multi-layer concrete beam transition elements were used on both sides of the joint to simulate the plastic ammonium zone at the beam end. Youssef [9] presented a refined component model using twelve concrete and steel springs and two diagonal axial springs for simulating the effects of bond-slip and crush of concrete. Although the model considers all factors, many assumptions are introduced in the modeling process, and the calculation is too large and the calculation efficiency is low.

The “super-node” element model proposed by Lowes et al. [10] and Mitra et al. [11] has comprehensive consideration factors, a reasonable mechanical model, and moderate calculation amount of numerical simulation, and can be used to simulate local inelastic deformation of nodes [12,13,14,15,16,17,18]. The “super-node” element model has been incorporated into the “Opensees” [19] structural analysis platform, which is called a beam-column joint element (BCJE) model. Zhao et al. [20] checked the characteristics of hysteretic curves at the loading end and the calculation results of inelastic deformation of two types of joints based on the test data of six intermediate joints. Shafaei et al. [21] used the BCJE model to simulate and analyze the nonlinear response of reinforced concrete frame structures under strong earthquakes. Tang [22] used this model to simulate reinforced concrete frames and steel-reinforced concrete frames. Xie et al. [23] simulated the low cyclic test of beam-column joints and verified that the simulation results were good. Shin and Lafavef [24] used the beam–column joint model to simulate and analyze the SL series reinforced by concrete beam–column joint tests. Based on the modified oblique compression field theory, the shear block components of the joint model were determined. The results showed that the joint model could well simulate the shear deformation of the joint area under cyclic loads and reflect the hysteretic performance of frame joints.

However, the tensile strength of plain concrete is very low, and its brittleness increases with the increase in strength grade. Adding randomly distributed steel fibers into concrete can effectively slow down the expansion of micro cracks and the generation and development of macro cracks in concrete, improve the tensile strength of concrete [25,26,27,28,29,30,31,32], reduce the brittleness of concrete, and achieve the purpose of strengthening and toughening [33,34,35,36,37,38]. In beams, the addition of steel fibers improves the concrete’s diagonal tension capacity, leading to increased shear resistance, which can promote flexural failure and ductility [39,40,41]. Tadepallil [42] made two types of hooked fibers and a twisted steel fiber as reinforcements in beam samples and studied the influence of the size, shape, and dosage of steel fibers on the mechanical properties of concrete. The test results showed that adding steel fiber into concrete could significantly improve the bending strength, bending toughness, and bending performance, while slightly improve the compressive strength and elastic modulus. Yang et al. [43] carried out semicircle three-point bending tests of ordinary asphalt concrete and basalt fiber asphalt concrete. The results showed that adding basalt fiber could improve the bending and tensile properties of the specimens, and played a role in toughening and cracking resistance in the fracture stage. Ganesan [44] discussed the influence of steel fiber content on the seismic performance of steel fiber reinforced concrete (SFRC) beam-column joints (BCJs) through a low cyclic loading test. The steel fiber content was 0, 0.25%, 0.50%, 0.75%, and 1%, respectively. It was found that the ductility, energy consumption, and shear capacity of beam-column joints also increased with the increase in the steel fiber volume ratio. Kytinou [45] studied the hysteretic behavior of steel fiber reinforced concrete slender deep beams. The results showed that SFRC beams exhibit enhanced cyclic performance in terms of residual stiffness, bearing capacity, deformation, energy dissipation capacity, and cracking performance. Shannag [46] found that compared with reinforced concrete BCJs, the energy dissipation capacity of high–performance fiber reinforced concrete BCJs increased by 20 times, and the stiffness degradation decreased by two times.

Based on the above background, according to the mechanical characteristics of steel fiber reinforced concrete beam-column joints (hereafter called SFRC–BCJs), a suitable analysis model is proposed by using Opensees software (version 2.3.0), and the hysteretic behavior under low cyclic loading is analyzed. Eight groups of SFRC–BCJs were simulated, and the rationality of numerical simulation was verified by comparing the experimental results with the simulation results. The effects of steel fiber volume ratio, stirrup ratio, and axial compression ratio on the seismic behavior of SFRC–BCJs are further analyzed. Finally, a formula for predicting ultimate shear capacity is derived, which is based on the steel fiber volume ratio, stirrup amount, and axial compression ratio.

## 2. Experimental Introduction

**Figure 1:**
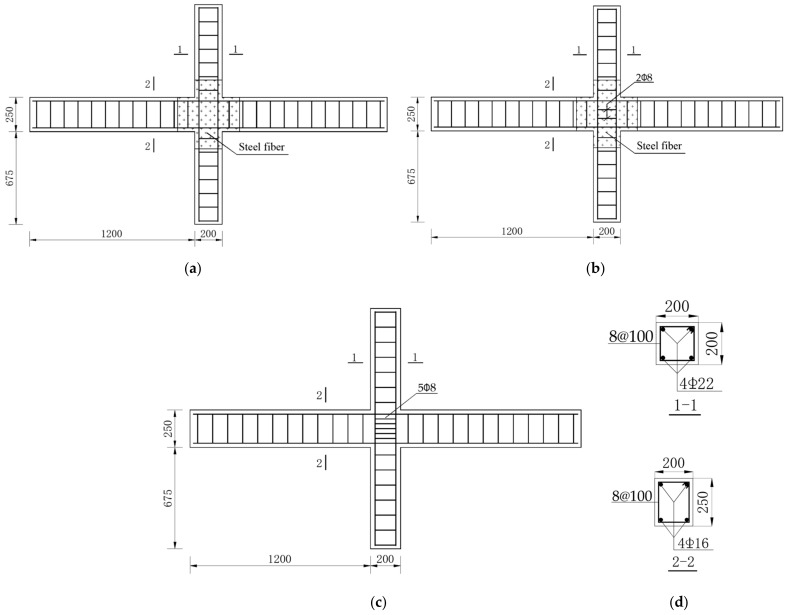
Geometry and reinforcement configuration of specimens: (**a**) BCJ1–0, BCJ1–1, BCJ1–2, BCJ3–2 and BCJ3–3; (**b**) BCJ2–2 and BCJ3–1; (**c**) BCJ5–1; (**d**) Reinforcement of specimen (units: mm).

To study the seismic behavior and energy dissipation performance of SFRC–BCJs, 13 groups of specimens have been manufactured by Shi [47]. In this paper, eight groups of specimens were selected and numerically simulated by Opensees. Figure 1 illustrates the detailing of geometry and reinforcement configuration in the BCJs, where 2 and ϕ8 in the symbol 2ϕ8 indicated the number and diameter of stirrups in the joint core area according to the related reference [47], 8 and @100 in the symbol 8@100 referred the number of stirrups, and horizontal or longitudinal spacing of stirrups in mm, respectively. The mechanical properties of reinforcing bars adopted in this study are listed in Table 1.

**Figure 2:**
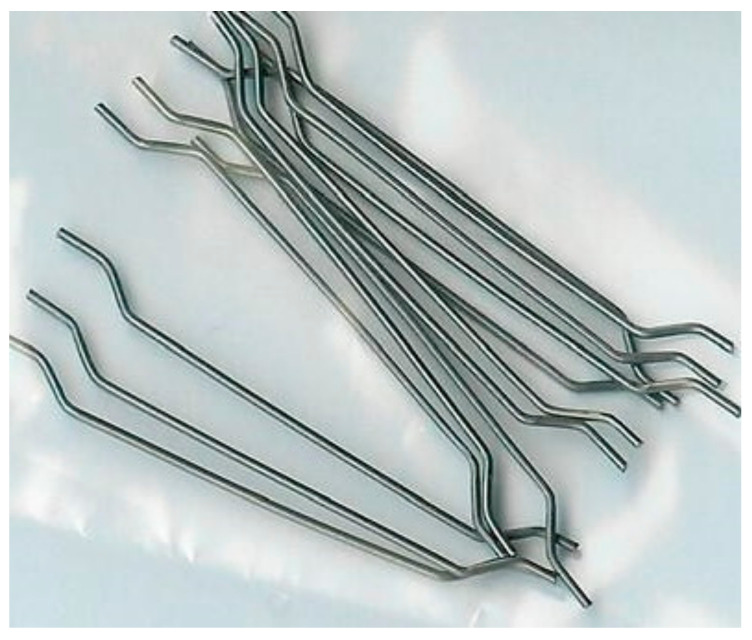
Steel fiber.

The parameters of BCJs are listed in Table 2. As shown in Figure 2, the aspect ratio of the fiber used was equal to *l_f_*/*d_f_* = 35 mm/0.55 mm = 64, and the nominal yield tensile strength was 1345 MPa. The mixed proportion of materials is summarized in Table 3. More information on the SFRC–BCJs could be referred in Shi [47].

**Figure 3:**
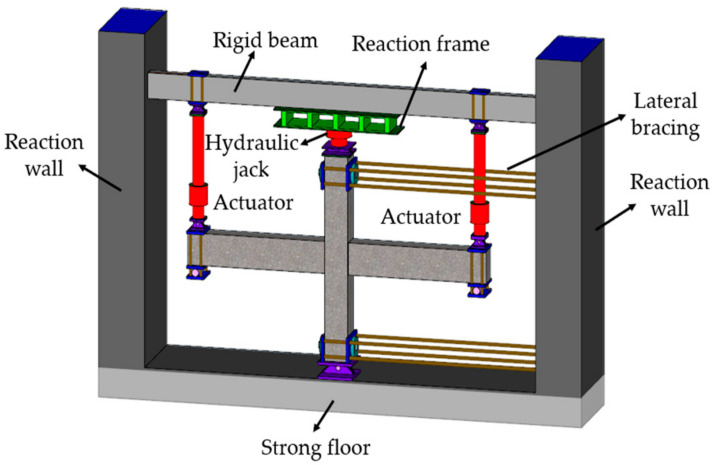
Schematic diagram of joint loading device.

**Figure 4:**
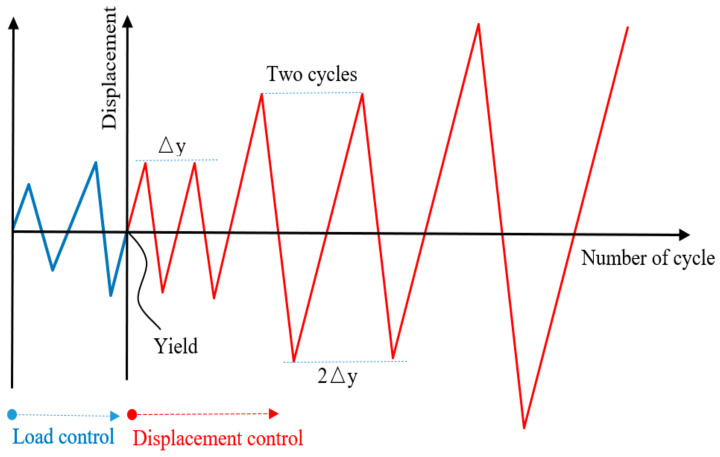
Loading system.

A schematic view of the loading apparatus is shown in Figure 3. The set axial pressure was applied on the top of the column with a hydraulic jack and kept constant. Then, a cyclic load was loaded at the beam ends by a hydraulic servo actuator. The loading system adopted the mixed control of load and displacement, as shown in Figure 4. Before yielding, the specimen was loaded by a load control, with 75% and 100% of yield load cyclic loading, once per stage. After yielding, the specimen was loaded by displacement control with the multiple of yield displacement as the step difference, and the displacement amplitude of each step was cycled twice. When the load was reduced to about 85% of the ultimate load, the test was stopped.

## 3. Finite Element Model

### 3.1. Element Model

#### 3.1.1. Nonlinear Fiber Beam–Column Element

**Figure 5:**
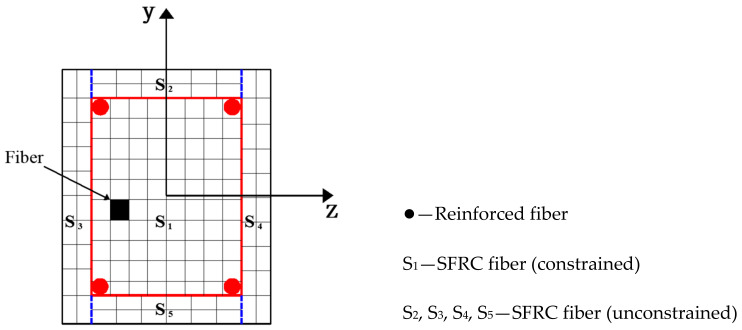
Fiber section model.

In this paper, the distributed plasticity element of the nonlinear beam–column element in the bar system model was adopted, which is provided by users in Opensees standard solver. The stiffness of beam and column elements can be changed along the length of the bar, and multiple control sections can be set on the length of the element. The beam and column element section restoring force model–fiber section model was adopted in the control section, as shown in Figure 5. The fiber section model discretizes each longitudinal control analysis section of the element into several longitudinal small elements, including confined concrete fibers, unconstrained concrete fibers, and reinforced fibers. Practice shows that when the number of fibers divided into sections reaches a certain amount, the error caused by the numerical integration method will no longer be significant, so the number of fibers divided into sections is not as good as possible. For the common rectangular section in plane problem analysis, enough calculation accuracy can be obtained when the number of fibers reaches about 40. Based on the assumption of the plane section, the fiber section model did not consider the effect of joint shear deformation and longitudinal reinforcement bond slip. At the same time, it is assumed that each fiber on the cross-section was under a uniaxial stress–strain state and a uniaxial stress–strain relationship which is more consistent with the stress state of the cross-section was obtained by considering the effect of stirrup constraint through appropriate modification [48,49,50].

#### 3.1.2. Beam–Column Joint Element

**Figure 6:**
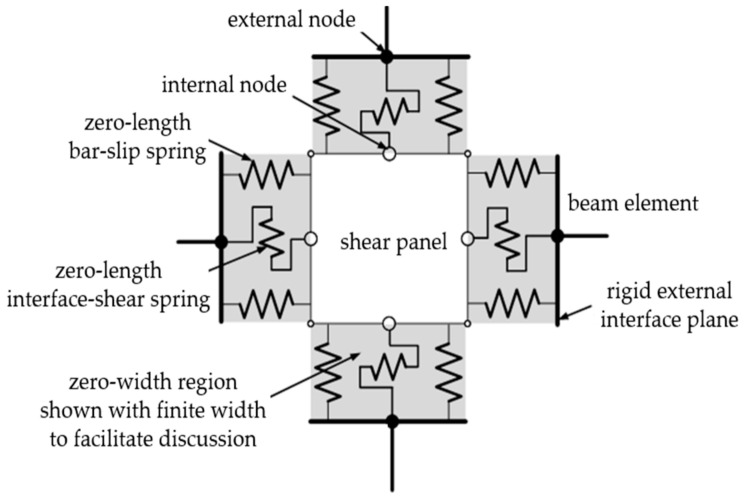
Beam–column joint element.

To reasonably reflect the stress mechanism of BCJs, the Opensees help file [51] introduced a joint element model with eight bar–slip springs, a shear–panel, and four interface–shear springs (see Figure 6). The features of the springs in this model were determined on the basis of test results. The model can further improve the simulation accuracy, and simulate the failure modes including anchorage failure, shear failure at the beam–column interface, and shear failure at the core area. Failure modes can be simulated by the following three component models: (1) One shear block component simulates the degradation of joint strength and stiffness caused by a shear failure in the core area of joints; (2) four interface shear components simulate the degradation of shear transfer ability at the interface around the joint; (3) eight slip components of reinforcing bars simulate the degradation of stiffness and strength of joints caused by the bond degradation of longitudinal reinforcing bars in the core area of BCJs.

**Figure 7:**
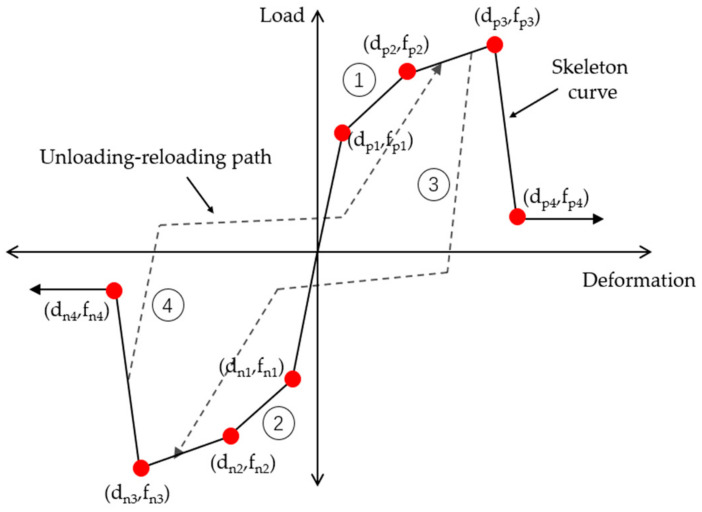
Generalized one-dimensional load–deformation hysteretic response curve.

In this paper, the one–dimensional hysteretic load–deformation model (Pinching4 material model) is used to describe the response of joint concrete spring [51]. As shown in Figure 7, the Pinching4 material model is defined using a response envelope, an unload–reload path, and three damage rules that control the development of the response path. The skeleton line was multilinear, and the unloading–reloading curve was trilinear. Based on this hysteretic material model, the user can define the force–deformation relationship of steel slip component, shear block component, and interface shear component in the joint model through the geometric dimensions of specific beam–column joint specimens, reinforcement conditions, and material features of reinforcing bars and concrete, and then check it with the test data of related materials. When the one-dimensional pinched material model was used to simulate the hysteretic response of the shear block, the physical meaning of load and deformation in the material model was not clear, which can be expressed by bending the moment–curvature relationship or stress–strain relationship. Through the dimensional analysis of the stress balance of the beam–column joint element, it can be determined that the load is the bending moment in the core area of the joint, and the deformation was the shear deformation in the core area of the joint. Song [52] suggested that the bending moment in the core area of the joint can be obtained by multiplying the shear stress of the shear block by the joint volume. Therefore, to define the skeleton line of the one-dimensional pinched material model, the shear stress–shear strain relationship of the shear block should be obtained. The shear stress and shear strain of four key points should be selected to determine the feature points of the pinched material model. Among them, feature point 1 was the cracking point, which is the starting point of stiffness change in the whole shear stress–strain curve; feature point 2 was the yield point, which is the point where the stiffness decreased obviously in the whole shear stress–strain curve; feature point 3 was the ultimate bearing load point, which is the maximum stress point in the whole shear stress–strain curve; feature point 4 was the failure point, which corresponds to the point when the shear stress drops to 85% of the ultimate shear stress in the whole shear stress–strain curve. Skeleton curve parameters were generally calculated in real-time according to related problem attributes. In contrast, loading and unloading parameters and degradation criteria parameters can be determined according to the parameters recommended in [51].

#### 3.1.3. Constitutive Model of Concrete

Concrete02 [53] with linear tension softening was used to model SFRC. Compared with the Concrete01 model, this model simplifies the algorithm and considers the tensile properties of concrete. Before cracking, tensile concrete is usually considered to obey the linear elastic hypothesis. However, after cracking, concrete between two adjacent cracks still bears certain tensile stress due to the bonding effect, that is, the tensile stiffening effect of concrete. Therefore, the linear ascending section and linear descending section describe the linear elastic behavior of concrete before cracking and tensile stiffening behavior after cracking. It contains seven variables: *f*_c_′ is compressive strength; $\varepsilon_{0}$ is strain at maximum strength; *f_cu_* is crushing strength; $\varepsilon_{cu}$ is strain at crushing strength; *λ* is ratio between unloading slope at crushing strength and initial slope (default value 0.01); *f_t_*′ is tensile strength; *E*_0_ is elasticity modulus; and *E*_ts_ tension softening stiffness. These variables were determined based on the cyclic compressive test for SFRC, which can be briefly described as follows

(1) $$\begin{array}{l} E_{0}=2f_{c}^{\prime }/\varepsilon_{0}\\ \varepsilon_{0}=0.002K\\ f_{cu}=Kf_{c}^{\prime }[1-Z(\varepsilon_{cu}-\varepsilon_{0})]\\ K=1+\rho_{sv}f_{yh}/f_{c}^{\prime }\\ Z=\frac{0.5}{\varepsilon_{50u}-\varepsilon_{0}}\\ \varepsilon_{50u}=\frac{3+0.29f_{c}^{\prime }}{145f_{c}^{\prime }-1000}\\ \varepsilon_{cu}=0.004+0.003\rho_{sv}f_{yh}\\ f_{t}=0.6228\sqrt{f_{c}^{\prime }}\\ E_{\mathrm{ts}}=0.1E_{0} \end{array}$$

where $\varepsilon_{50u}$ represents the strain corresponding to the stress equivalent to 50% of the maximum concrete strength of unconfined concrete [54]; *K* represents the reinforcement coefficient to consider the influence of stirrup restraint effect on the strength and ductility of beam-column joint concrete [55].$\;\rho_{sv}$ is the volume ratio of transverse steel in the core area of concrete; $f_{yh}$ is the yield strength of transverse steel.

#### 3.1.4. Constitutive Model of Reinforcement

The reinforced material constitutive model adopted in this paper is the Steel02 model in the Opensees platform, which is a modified model [56,57] based on the reinforced constitutive model proposed by Menegotto and Pinto [58], considering the influence of isotropic strain hardening of reinforced materials. This model has high calculation efficiency and can better reflect the basic mechanical properties of reinforced materials under repeated loading and unloading conditions and better reflect the Bauschinger effect.

The parameters that need to be determined artificially in the Steel02 model include yield strength *f*_y_, elasticity modulus *E*, and strain hardening rate *B* when reinforcing bars enter the strengthening stage after yielding. The curve transition shape is determined by the parameter *R*, which is called the curvature coefficient of the transition curve, and the curvature radius of the curve increases with the decrease in *R*, and its specific value can be calculated according to the following formula:

(2) $$R=R_{0}-\frac{a_{1}\xi }{a_{2}+\xi }$$

In the analysis of this paper, the ξ  is the maximum strain-related parameter in strain history, and its value will be updated every time the strain is reversed. *R*_0_ is the curvature coefficient of the curve under initial loading. The *a*_1_ and *a*_2_ are curvature degradation coefficients under reciprocating loading. The parameter considering isotropic strain hardening adopts the default value of Opensees.

#### 3.1.5. Analysis Module

In this paper, the Plain method was selected as the two-dimensional structural constraint processing module. Numbers were sorted by RCM optimized nodes. BandGeneral was used for rectangular bandwidth processing. Krylov–Newton was used for iterative calculations considering the P-delta effect. In order to facilitate convergence, the energy criterion was used, the tolerance was set to $5\times 10^{-8}$, and the maximum iteration step was set to 100.

### 3.2. Applicability Analysis of Beam–Column Joint Element Model

**Figure 8:**
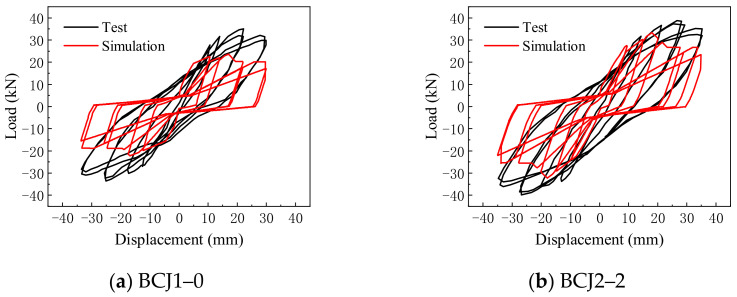
Comparison of hysteretic curves.

The typical SFRC beam–column joint specimens BCJ1–0 and BCJ2–2 are numerically simulated used the beam–column joint element model. When the finite element model was established, the beam and column adopted the nonlinear beam–column element model. Its section adopted the fiber section. The beam–column joint element model was adopted for beam–column joints. The Concrete02 constitutive model was adopted for SFRC. The Steel02 constitutive model was adopted for reinforcement. The hysteretic curves obtained by simulation were compared with the experimental results, as shown in Figure 8.

From the comparison results, it can be seen that the hysteresis curve obtained by simulation was quite different from the test results, the hysteresis loop was too pinched, the energy consumption and ductility were too small, and the yield load and ultimate load were significantly smaller than the test results, which indicated that there was an obvious deviation in simulating SFRC–BCJs by the beam–column joint element model. The main reason is that the component parameters of the beam–column joint element model were determined according to the calculation method proposed by Mitra [59] based on the test results of reinforced concrete beam–column joints. The influence of steel fiber content on component parameters of the beam–column joint element model was not considered.

### 3.3. Improvement of Beam–Column Joint Element Mode

#### 3.3.1. Constitutive Model of Reinforced Bond–Slip Spring

**Figure 9:**
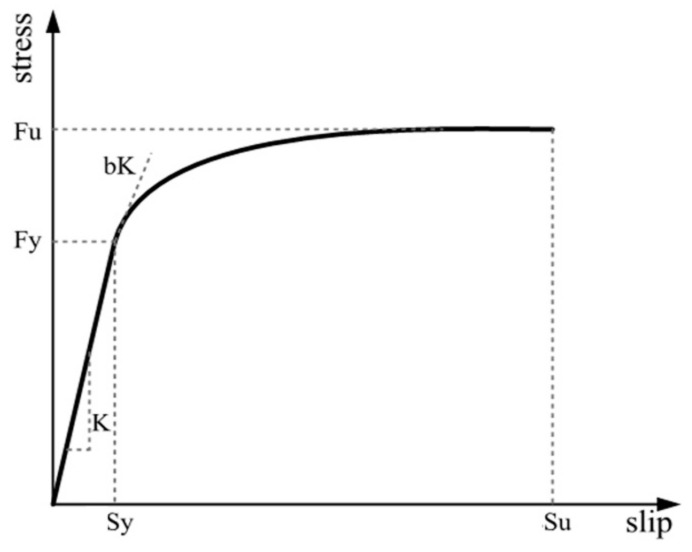
Bond_SP01 model reinforcement stress–slip envelope curve.

Considering the influence of steel fiber on the bond performance between steel bar and concrete, the Bond_SP01 material model is used to simulate the bond-slip component of reinforcing bars in this paper, and the stress–slip relationship curve of reinforcing bars is shown in Figure 9.

In Figure 9, *F*_y_ and *F*_u_ represent the yield strength and ultimate strength of reinforcing bars, *S*_y_ is rebar slip at member interface under yield stress, and *S*_u_ is rebar slip at the loaded end at the bar fracture strength. The hardening rate *b* of the hardening starting point on the slip stress relation curve of monotonically loaded reinforcing bars is 0.4. The pinching coefficient *r* of the slip stress hysteretic curve of cyclically loaded reinforcing bars is 0.6.

In Figure 9, considering the influence of steel fiber incorporation on the bond-slip between reinforcement and concrete, $S_{y}$ adopted in this paper is based on the formula recommended by Harajli [60], which can be expressed as

(3) $$S_{y}=S_{1}e^{1.8[{\left(u_{\mathrm{sp}}/u_{m}\right)}^{2}-1)]}$$

where

(4) $$S_{1}=0.15c_{0}$$

(5) $$u_{\mathrm{sp}}=0.78\sqrt{f_{c}}{\left(\frac{c+0.45cV_{f}L_{f}/D_{f}}{d_{b}}\right)}^{2/3}$$

(6) $$u_{m}^{}=2.57\sqrt{f_{c}}$$

where $c_{0}$ is the net distance of longitudinal stressed reinforcing bars, *u*_sp_ is yield bond stress, *u*_m_ is the maximum bond stress, *c* is the minimum concrete cover thickness, *f*_c_ is the compressive strength of concrete, *V*_f_ is the volume ratio of steel fiber, *L*_f_*/D*_f_ is the aspect ratio of steel fiber, and *d_b_* is the diameter of longitudinal reinforcement.

Rebar slip at the loaded end at the bar fracture strength *S*_u_ can be computed as follows

(7) $$S_{u}=35S_{y}$$

#### 3.3.2. Constitutive Model of Joint Shear Block

**Figure 10:**
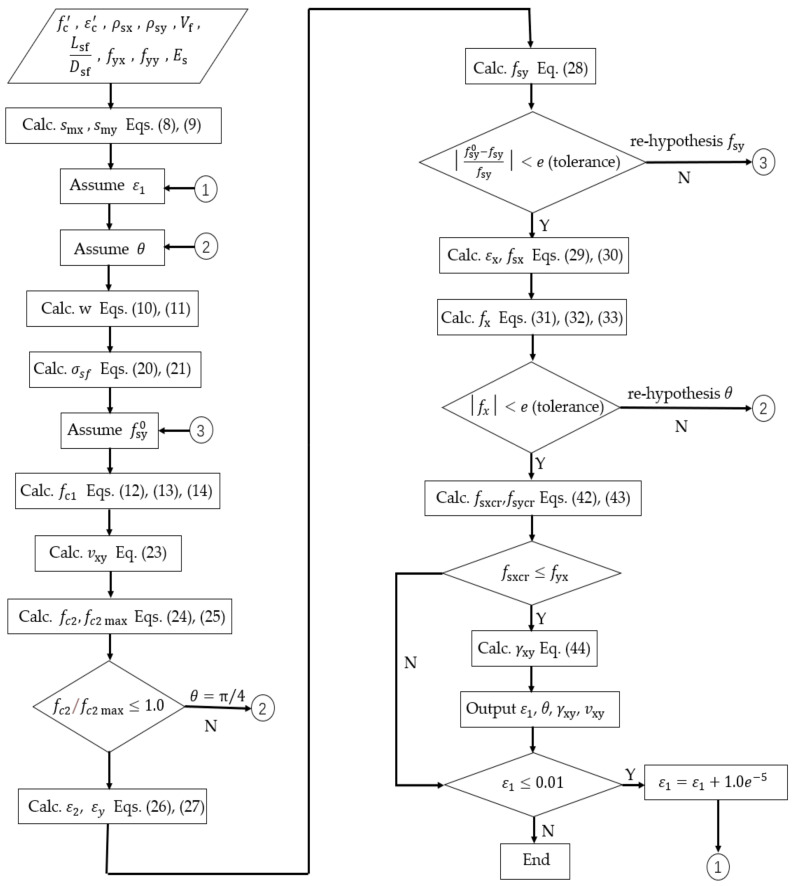
Flow chart showing an efficient algorithm.

Vecchio and Collins [61] have developed the pressure field theory and put forward the modified compression–field theory (MCFT), which establishes the constitutive relation of cracked concrete and can consider the influence of tensile properties at cracks on compressive strength. In this paper, considering the impact of steel fiber on the shear block, according to the equilibrium condition, compatibility condition, and constitutive relation of steel bar and concrete material proposed by MCFT, the shear stress–shear strain constitutive relation of SFRC in the core area of joint under monotonic pure shear load is predicted by calculation [62]. A set of solution procedures is proposed, as shown in the flow charts in Figure 10. The calculation process is as follows:

Step 1—Determine the crack control feature value of longitudinal reinforcement and stirrup.

(8) $$s_{\mathrm{mx}}=1.5\times s_{x}$$

(9) $$s_{\mathrm{my}}=1.5\times s_{y}$$

where $s_{x}$ and $s_{y}$ are the maximum spacing between longitudinal reinforcement and stirrup, respectively.

Step 2—given the principal tensile strain $\varepsilon_{1}$ of concrete.

Step 3—assuming the azimuth angle *θ* of the principal compressive stress.

Step 4—calculate average crack width *w* using Equations (10) and (11).

(10) $$s_{\theta }=\frac{1}{\frac{\sin\theta }{s_{\mathrm{mx}}}+\frac{\cos\theta }{s_{\mathrm{my}}}}$$

(11) $$w=\varepsilon_{1}s_{\theta }$$

where *s_θ_* is the average crack spacing in the core area of SFRC–BCJs.

Step 5—assume the stress $f_{\mathrm{sy}}^{0}$ of reinforcement in Y direction;

Step 6—the average tensile stress $f_{c1}$ of concrete is calculated by Equations (12)–(14).

When $E_{1}\leq E_{\mathrm{cr}}$

(12) $$f_{c1}=E_{c}\varepsilon_{1}$$

When $E_{1}>E_{\mathrm{cr}}$, for normal concrete,

(13) $$f_{c1}=\frac{f_{\mathrm{cr}}}{1+\sqrt{500\varepsilon_{1}}}$$

and for SFRC [63],

(14) $$f_{c1}=\frac{f_{\mathrm{cr}}+\beta \sigma_{\mathrm{tu}}}{1+\beta }$$

among them

(15) $$E_{c}=\frac{2f_{c}^{\prime }}{\varepsilon_{c}^{\prime }}$$

(16) $$\beta =\sqrt{\frac{\varepsilon_{1}-\varepsilon_{\mathrm{cr}}}{0.005}}$$

where $f_{c}^{\prime }$ is the compressive strength of cylinder (negative); $\varepsilon_{c}^{\prime }$ is the strain at compressive strength of cylinder (Generally taken as –0.002);$\;f_{\mathrm{cr}}=0.33\sqrt{f_{c}^{\prime }}$, $\;f_{cr}$ is the cracking stress of concrete,$\;\varepsilon_{\mathrm{cr}}=f_{\mathrm{cr}}/E_{c}$; and $\varepsilon_{\mathrm{cr}}$ is the cracking strain of concrete.

Equations (12)–(14) should satisfy Equation (17).

(17) $$f_{c1}\leq v_{ci\max}(0.18+0.3k^{2})\tan\theta +\rho_{sy}(f_{yy}-f_{sy}^{0})+\sigma_{sf}$$

among them

(18) k=1.64−1/tanθ≥0

(19) $$\rho_{sy}=\frac{A_{sy}}{A_{c}}$$

where $\rho_{\mathrm{sy}}$ is the Y–direction reinforcement ratio;$\;A_{c}$ is the Section area of concrete column;$\;\sigma_{\mathrm{sf}}$ is

the tensile stress of steel fiber per unit area of crack surface,$\;\sigma_{\mathrm{sf}}$, can be calculated as

(20) $$\sigma_{sf}=k_{sf}\tau_{sf}v_{sf}\frac{l_{sf}}{d_{sf}}$$

where the effective distribution coefficient of steel fiber [64],$\;k_{\mathrm{sf}}$, can be expressed as

(21) $$k_{sf}=\frac{\tan^{-1}(3.5w/l_{sf})}{\pi }{\left(1-\frac{2w}{d_{sf}}\right)}^{2}$$

where $\tau_{\mathrm{sf}}$ is the bond stress between steel fiber and concrete; $\tau_{\mathrm{sf}}=2.5f_{\mathrm{ct}}$, ($f_{ct}$ is the tensile strength of concrete);$\;v_{\mathrm{sf}}$ is the volume ratio of steel fiber in the core area of SFRC–BCJs; and$\;l_{\mathrm{sf}}/d_{\mathrm{sf}}$ is the length–diameter ratio of steel fiber.

(22) $$\nu_{ci\max}=\frac{\sqrt{-f_{c}^{\prime }}}{0.31+24w(\alpha +16)}$$

where a is the maximum particle size of concrete coarse aggregate;$\;v_{\mathrm{ci}\;\max}$ is the maximum shear force that can be borne on a crack with a given width.

Step 7—the shear stress $v_{\mathrm{xy}}$ of the element relative to the *X* and *Y* axes is calculated by the equilibrium condition.

(23) $$\nu_{xy}=(f_{c1}+\rho_{sy}f_{sy}^{0}+\sigma_{sy}\sin^{2}\theta )/\tan\theta$$

Step 8—calculate compressive stress$\;f_{c2}$.

(24) $$f_{c2}=f_{c1}-\nu_{xy}(\tan\theta +1/\tan\theta )$$

Step 9—calculate the maximum compressive stress.

(25) $$f_{c2\max}=\frac{f_{c}^{\prime }}{0.8-0.34\varepsilon_{1}/\varepsilon_{c}^{\prime }}$$

Step 10—check that $f_{c2}/f_{c2\;\max}\leq 1.0$. If greater than 1.0, then solution is not possible; return to Step 3 and choose *θ* closer to 45 deg or return to Step 2 and choose a lower $\varepsilon_{1}$.

Step 11—calculate compressive strain $\varepsilon_{2}$.

(26) $$\varepsilon_{2}=\varepsilon_{c}^{\prime }(1-\sqrt{1-f_{c2}/f_{c2\max}})$$

Step 12—calculate the strain $\varepsilon_{y}$ in the *Y* axis direction.

(27) $$\varepsilon_{y}=\frac{\varepsilon_{1}+\varepsilon_{2}\tan^{2}\theta }{1+\tan^{2}\theta }$$

Step 13—calculate the stress $f_{\mathrm{sy}}$ of *Y*–direction reinforcement.

(28) $$f_{sy}=E_{s}\varepsilon_{y}$$

Step 14—check if $f_{\mathrm{sy}}$ calculated agrees with estimated $f_{\mathrm{sy}}^{0}$. If not, return to Step 5 with new estimate of $f_{\mathrm{sy}}^{0}$.

Step 15—calculate the strain $\varepsilon_{x}$ of longitudinal reinforcement (*X* axis direction).

(29) $$\varepsilon_{x}=\varepsilon_{1}+\varepsilon_{2}-\varepsilon_{y}$$

Step 16—calculate the stress $f_{\mathrm{sx}}$ of reinforcement in *X* direction.

(30) $$f_{sx}=E_{s}\varepsilon_{x}$$

Step 17—calculate the x–direction stress $f_{x}$ of the element.

(31) $$f_{x}=f_{c1}-v_{xy}/\tan\theta +\rho_{sx}f_{sx}+\sigma_{sf}\sin^{2}\theta$$

among them

(32) $$\rho_{sx}=\frac{A_{sx}}{A_{b}}$$

(33) $$A_{b}=h_{j}b$$

In this paper, it is considered that the longitudinal reinforcement of the beam has no restraint function of vertical stirrups, so it has no restraint function of shear deformation, and its contribution to the shear resistance of the joint area can be ignored. Therefore, when calculating $\rho_{\mathrm{sx}}$, $A_{\mathrm{sx}}$ can be taken as the cross-sectional area of the hoop limb of the horizontal stirrup in the joint along the force direction. *A_b_* represents section area of the concrete beam.

Step 18—check if $f_{x}$ is equal to 0. If not, return to Step 3 and make new estimate of θ.

Step 19—calculate stresses on crack $v_{\mathrm{ci}}$ and $f_{\mathrm{ci}}$.

(34) $$\Delta f_{ci}=f_{c1}-\rho_{sy}(f_{yy}-f_{sy})$$

If $\Delta f_{\mathrm{ci}}\leq 0$, then $v_{\mathrm{ci}}=0$ and $f_{\mathrm{ci}}=0$. Go to Step 20.

If $\Delta f_{\mathrm{ci}}>0$, then

(35) $$C=\frac{\Delta f_{\mathrm{ci}}}{\tan\theta }-0.18v_{ci\max}$$

If C≤0, then

(36) $$f_{\mathrm{ci}}=0$$

(37) $$\nu_{\mathrm{ci}}=\Delta f_{\mathrm{ci}}/\tan\theta$$

Otherwise

(38) $$A=0.82/v_{\mathrm{ci}\;\max}$$

(39) $$B=\frac{1}{\tan\theta }-1.64$$

(40) $$f\mathrm{ci}=(-B-\sqrt{B^{2}-4AC})/(2A)$$

(41) $$\nu_{\mathrm{ci}}=(f_{\mathrm{ci}}+\Delta f_{c1})/\tan\theta$$

Step 20—calculate the reinforcement stress at crack $f_{\mathrm{sxcr}}$ and $f_{\mathrm{sycr}}$.

(42) $$f_{\mathrm{sxcr}}=f_{\mathrm{sx}}+(f_{c1}+f_{\mathrm{ci}}-\sigma_{\mathrm{sf}}+v_{\mathrm{ci}}\cot\theta )/\rho_{\mathrm{sx}}$$

(43) $$f_{\mathrm{sycr}}=f_{\mathrm{sy}}+(f_{c1}+f_{\mathrm{ci}}-\sigma_{\mathrm{sf}}-v_{\mathrm{ci}}\tan\theta )/\rho_{\mathrm{sy}}$$

Step 21—check that reinforcement can carry stress at crack. If $f_{\mathrm{sxcr}}\geq f_{\mathrm{yx}}$, assume a lower $f_{c1}$ and return to Step 7.

Step 22—Calculate the shear strain $\gamma_{\mathrm{xy}}$.

(44) $$\gamma_{\mathrm{xy}}=2(\varepsilon_{x}-\varepsilon_{2})/\tan\theta$$

In order to obtain a complete unit reaction, these calculations are repeated within a certain range of $\varepsilon_{1}$. $\varepsilon_{1}$ increases from less than the cracking strain until the maximum shear stress is obtained.

**Figure 11:**
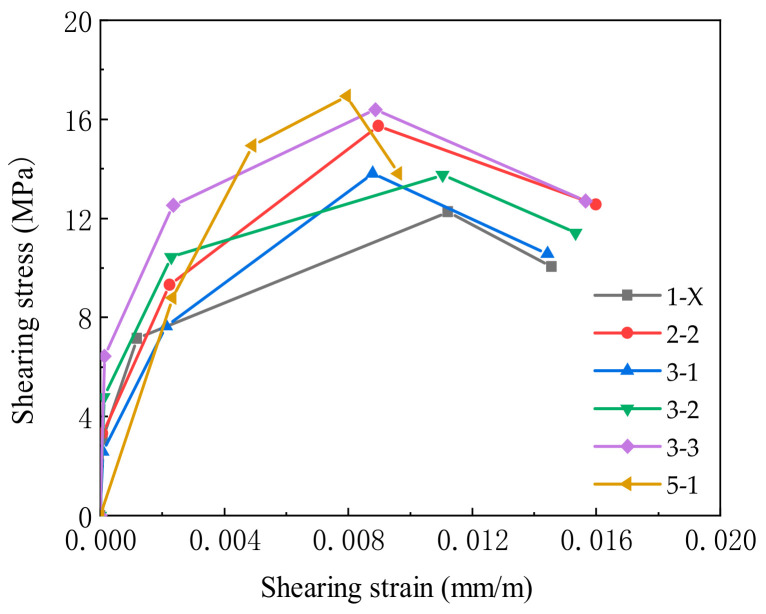
Selection of key points of shear block components in the joint core area (note: 1–X includes BCJ1–0, BCJ1–1, and BCJ1–2).

Figure 10 gives the flow chart showing an efficient algorithm. By inputting the compressive strength of SFRC, the strain at the compressive strength, the crack control feature values of reinforcing bars in *X* and *Y* directions, the stirrup ratio and reinforcement ratio in *X* and *Y* directions, the volume ratio of steel fibers, and the ratio of length to diameter, etc., the shear stress–shear strain relationship curve in the joint area shown in Figure 11 can be obtained. Finally, the key points are selected, and their coordinates are substituted into the Pinching4 material relation constitution.

After completing the definition of the shear stress–strain skeleton curve of the shear block in the core area, it is necessary to define its hysteretic rules. Stevens [65] developed on the basis of MCFT, which enabled the model to simulate the pinching features of hysteresis loops, which was caused by the opening and closing of cracks in reinforced concrete joints. This feature can be reflected by the definition of the reloading and unloading paths and the damage parameters. The parameters are as follows: the initial shear stress of reloading is 15% of the maximum shear stress reached in this cycle; the initial shear deformation of reloading is 25% of the maximum shear strain achieved in this cycle; the initial stress of unloading is taken as 0; Unloading stiffness degradation parameter:$\alpha_{1}=0.2,\alpha_{2}=0,\alpha_{3}=0.12,\alpha_{4}=0,limit=0.6$; reload stiffness degradation parameter:$\;\alpha_{1}=0.2$,$\;\alpha_{2}=0$,$\;\alpha_{3}=0.51$,$\;\alpha_{4}=0$, limit=0.4; stiffness degradation parameter:$\;\alpha_{1}=1.11$,$\;\alpha_{2}=0$,$\;\alpha_{3}=0.319$,$\;\alpha_{4}=0$, limit=0.125.

## 4. Numerical Result Analysis

The finite element analysis software Opensees was used to simulate the low–cycle reciprocating horizontal loading test of the eight BCJs as mentioned above. Considering the applicability of the model, the numerical simulation of FRCJ1, FRCJ2 [66], JZ2, and JZ3 [67] specimens of SFRC–BCJs was further carried out. By comparing hysteretic curves, skeleton curves, energy dissipation, and stiffness degradation of each specimen, the rationality and accuracy of the numerical modeling method proposed in this paper were verified.

### 4.1. Hysteretic Curve

**Figure 12:**
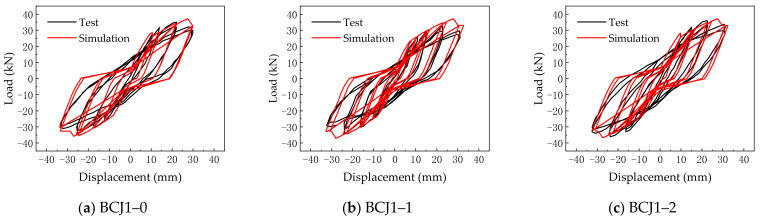

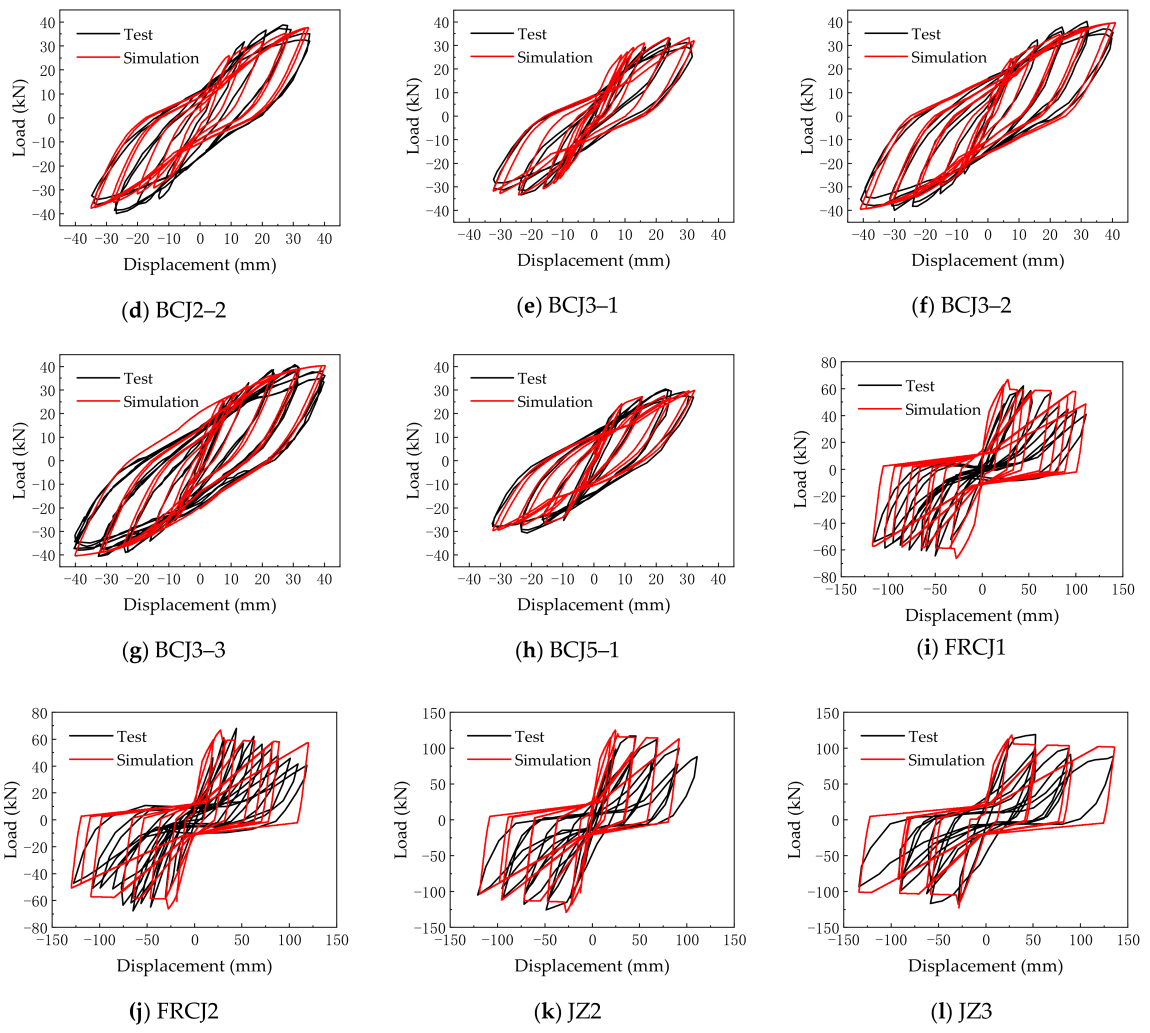
Hysteretic curve.

Figure 12 gives the comparisons of the load–displacement hysteretic curves between simulated and experimental results. As shown in Figure 12, it can be seen that the overall change trend of the theoretical and experimental hysteresis curves of each sample is the same, and the hysteresis curves are relatively complete. In addition, the pinch effect, strength degradation, and stiffness degradation of hysteretic curves are in good agreement with the experimental results. It indicated that it is feasible to apply the parameter calculation method proposed in Section 3.3 to SFRC–BCJs.

### 4.2. Skeleton Curve

**Figure 13:**
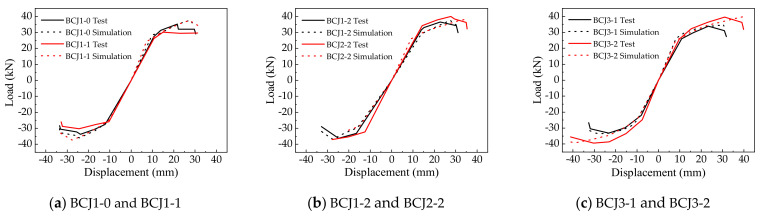

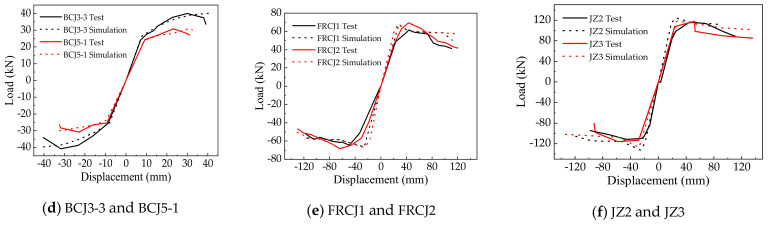
Skeleton curve.

Figure 13 gives the loading end load–displacement skeleton curves of twelve SFRC–BCJs. The load–displacement skeleton curves obtained by experiment and simulation have good symmetry about the origin. The skeleton curve of twelve SFRC–BCJs could be divided generally into three stages: elastic stage, strengthening stage, and degradation stage. After reaching the ultimate load, the curve decline rate of the load–displacement skeleton curve changed slowly. There was a long strength decline section, which behaved a better ductility of SFRC–BCJs. Comparative test and simulated skeleton curve, the skeleton curve was in good agreement, with an obvious yield load point and ultimate load point. The skeleton curve of numerical simulation showed an upward trend in the later period, mainly because the fatigue effect of reinforcement under low cyclic loading was not considered.

Table 4 lists the comparison results of the feature points between the test and simulation results. The ratios (T/S) are values of test results divided by the corresponding simulation results, where *P*_y_, *P*_m_, and *P*_u_ are the yield load, ultimate load, and failure load, respectively. As can be shown from Table 4, the discrepancies of the yield load, ultimate load, and failure load between experimental and corresponding simulate results are less than 9.1% for all the specimens. Besides, the average ratios on *P*_y_, *P*_m_, and *P*_u_ were 1.04, 0.98, and 0.94, and the corresponding COV (coefficients of variation) were 0.002, 0.0022, and 0.0002, respectively.

To sum up, the proposed numerical modeling approach in this study can predicate the seismic behavior of SFRC–BCJs with reasonable accuracy, which provides a basis for further study.

### 4.3. Energy Dissipation and Stiffness Degradation

**Figure 14:**
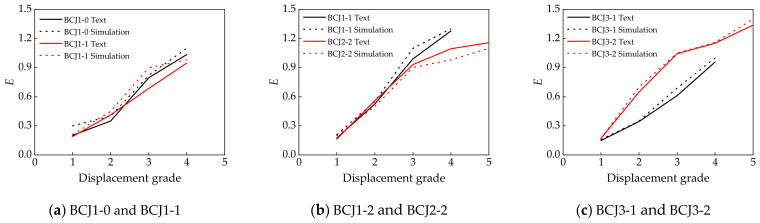

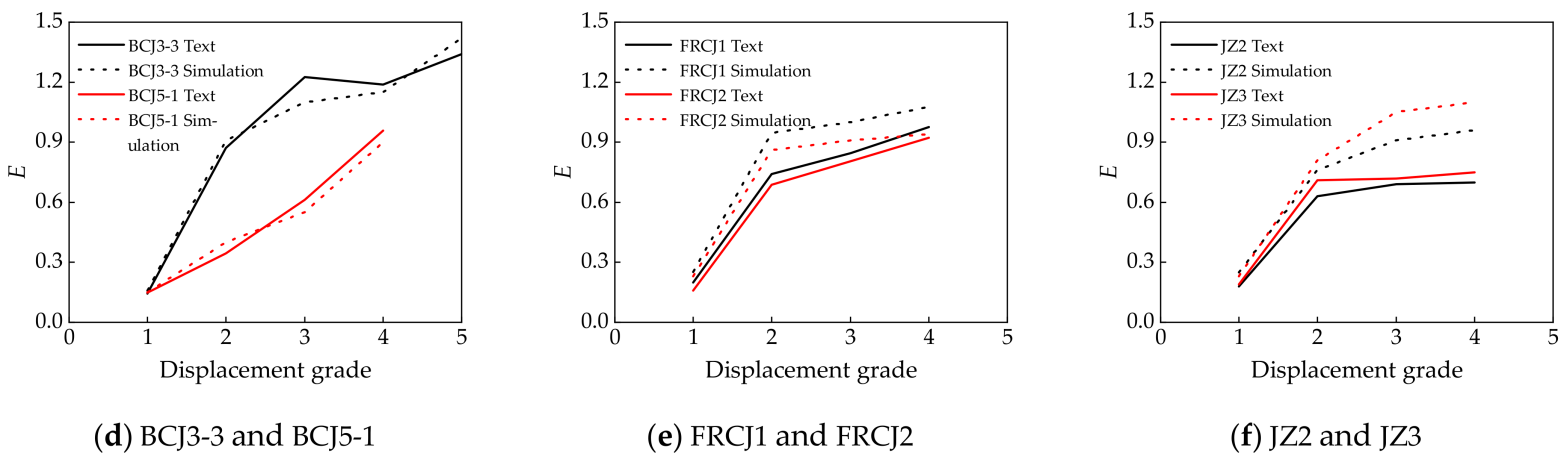
Relationship between energy dissipation coefficient and displacement grade.

**Figure 15:**
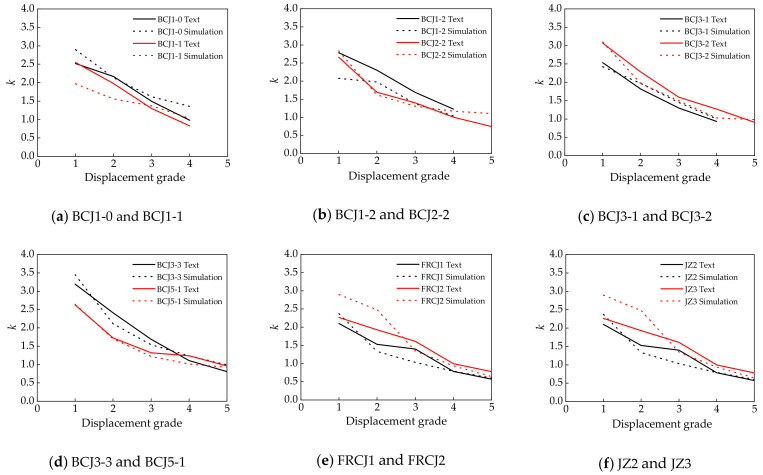
Relationship between stiffness degradation coefficient and displacement grade.

Figure 14 shows the comparisons of the energy dissipation curves between numerical and experimental results. The energy consumption of all the specimens can be evaluated by energy dissipation coefficient *E* [68], and the curve of the energy dissipation coefficient of each specimen is shown in Figure 14. With the increase in displacement grade, the energy dissipation coefficient increased. Figure 15 presents the comparisons of the stiffness degradation curves between numerical and experimental results. Stiffness degradation of specimens is another important index to measure the seismic capacity of joints, and it can be measured by the loop coefficient *k* [69]. It can be seen that the overall change trend of the theoretical and experimental curves of each sample was basically the same, and reasonable agreement between them was achieved.

## 5. Parameter Expansion Analysis

Based on the validated numerical modeling approach, the numerical modes were established to extensively investigate the effect of some key parameters on the seismic behavior of SFRC beam–column joints, including the steel fiber volume ratio and stirrup amount of the joint core area, as well as the axial compression ratio.

### 5.1. Steel Fiber Volume Ratio

**Figure 16:**
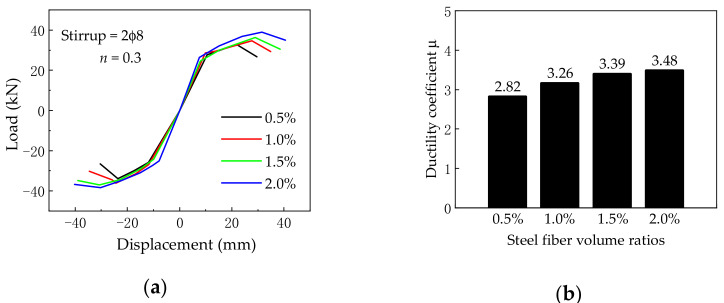
(**a**) Skeleton curves and (**b**) ductility coefficient under different steel fiber volume ratios.

Figure 16 gives the effect of steel fiber volume ratio on the load vs. displacement skeleton curves and ductility coefficient, where the steel fiber volume ratio rings from 0.5% to 2.0%, while others are kept the same. At first, the initial stiffness increased slightly with the increase in the steel fiber volume ratio. Secondly, as the steel fiber volume ratio increased from 0.5% to 2.0%, the ultimate load and ductility coefficients were improved by 16.2% and 23.4%, respectively. Therefore, increasing the steel fiber volume ratio helps to improve the seismic behavior of BCJs.

### 5.2. Stirrup Amount of Joint Core Area

**Figure 17:**
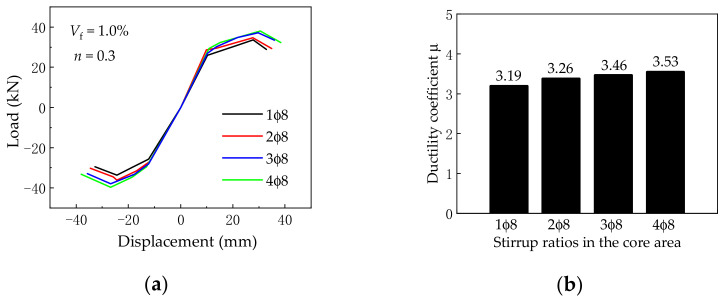
(**a**) Skeleton curves and (**b**) ductility coefficient under different stirrup ratios in the core area of joints.

Figure 17 presents the effect of different joint core area stirrup ratios on the load vs. displacement skeleton curves and ductility coefficient. The stirrups of the joint core area are 1ϕ8; 2ϕ8; 3ϕ8; respectively, and 4ϕ8; while others are kept the same. As can be seen from Figure 17, the initial stiffness almost remained unchanged. Besides, with the increase in the stirrup ratio in the core area of the beam–column joint, the ultimate load and ductility coefficient increase in the joint will increase accordingly. When the stirrup increases from 1ϕ8 to 4ϕ8, the ultimate load and ductility coefficient increase by 13.2% and 10.7%, respectively.

### 5.3. Axial Compression Ratio

**Figure 18:**
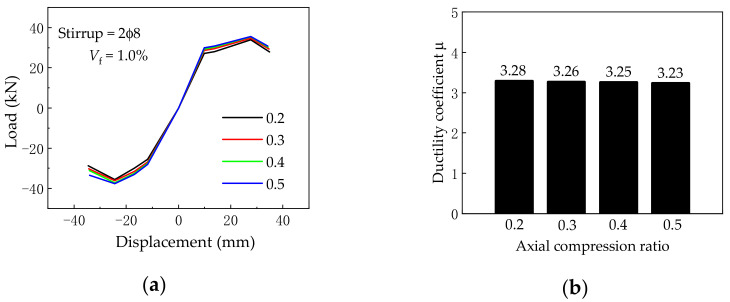
(**a**) Skeleton curves and (**b**) ductility coefficient under different axial compression ratios.

Figure 18 shows the effect of various axial compression ratios on the load vs. displacement skeleton curves and ductility coefficient, where the axial compression ratios are 0.2, 0.3, 0.4, and 0.5, while others are kept the same. It is found that the increase in the axial compression ratio leads to a slight increase in the initial tangent stiffness in the elastic stage. What is more, both the ultimate load, failure load, and ductility coefficient almost remained unchanged with the increase in the axial compression ratio. Therefore, the axial compression ratio has little effect on the seismic behavior of BCJs.

## 6. Ultimate Shear Capacity of SFRC–BCJs

The ultimate shear capacity of SFRC–BCJs can be provided by the steel fiber reinforced concrete and stirrup in the joint core area. Jiang [70] established the ultimate shear capacity formula of SFRC–BCJs, *V*_j_, which can be expressed as

(45) $$V_{j}=V_{\mathrm{cf}}+V_{s}$$

where $V_{\mathrm{cf}}$ is the shear bearing capacity of the steel fiber reinforced concrete; $V_{s}$ is the shear bearing capacity of the stirrup.

The research shows that steel fiber can enhance the shear strength of members and the tensile strength of the concrete matrix [71,72,73]. Hence, *V*_cf_ can be expressed by

(46) $$V_{\mathrm{cf}}=(\alpha_{1}+\alpha_{2}n)(1+\alpha_{3}v_{f})f_{c}h_{j}b_{j}$$

where $\alpha_{1}$, $\alpha_{2}$, and $\alpha_{3}$ are the influence coefficients; $h_{j}$ and $b_{j}$ are section height and width, respectively.

Refer to the calculation formula of shear capacity of reinforced concrete beam–column joints and consider the influence of uneven yield of stirrups in the core area of joints [74,75], $V_{s}$ can be written as follows

(47) $$V_{s}=\alpha_{4}f_{\mathrm{yv}}\frac{A_{\mathrm{sv}}}{s}(h_{0}-a_{s}^{\prime })$$

where $f_{\mathrm{yv}}$ is the tensile strength of stirrups; $A_{\mathrm{sv}}$ and *s* are stirrup cross–sectional area and spacing, respectively; *h*_0_ is the effective height of the core area of the joint; $a_{s}^{\prime }$ is the distance from the resultant force point of longitudinal reinforcement to the edge of concrete.

Substituting Equations (46) and (47) into Equation (45), the ultimate shear capacity *V*_j_ can be calculated as follows

(48) $$V_{j}=(\alpha_{1}+\alpha_{2}n)(1+\alpha_{3}v_{f})f_{c}h_{j}b_{j}+\alpha_{4}f_{\mathrm{yv}}\frac{A_{\mathrm{sv}}}{s}(h_{0}-a_{s}^{\prime })$$

According to the simulation results in this paper, the regression analysis of Equation (48) can be obtained:$\;\alpha_{1}=0.0764$, $\alpha_{2}=0.0082$, $\alpha_{3}=11.523$, $\alpha_{4}=0.90$. The formula for calculating the ultimate shear capacity of the core area of SFRC–BCJs can be expressed as

(49) $$V_{j}=(0.0764+0.0082n)(1+11.523v_{f})f_{c}h_{j}b_{j}+0.9f_{\mathrm{yv}}\frac{A_{\mathrm{sv}}}{s}(h_{0}-a_{s}^{\prime })$$

To verify the applicability of this formula, we selected the domestic experimental research literature on SFRC–BCJs, calculated the joint specimens with Equation (49), and compared the calculated values with the measured values. The specific parameters and comparison results are shown in Table 5, below.

**Figure 19:**
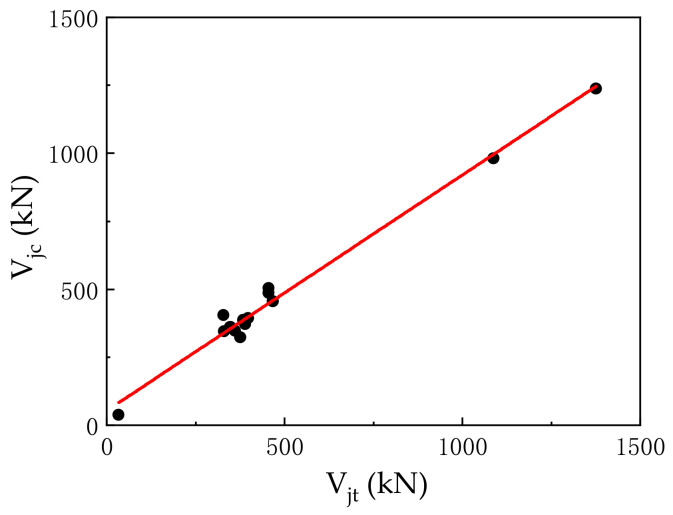
Regression analysis curve.

As shown in Table 5, the ultimate shear capacity obtained by solving the formula is in good agreement with the numerical value measured by the test. The ratio of the simulated value to the calculated value is about 0.812 to 1.163. From Table 5, it also can be seen that the average ratio between the experimental value *V***_jt_** and the calculated value *V*_jc_ is 0.997 with the COV of 0.094. Figure 19 shows the comparisons of regression analysis curve between the simulated and experimental results. There is no obvious dispersion between the calculated values and the measured values. The dispersion degree is low, proving the feasibility of using this formula to calculate the ultimate shear capacity of the SFRC beam–column joint core area.

## 7. Conclusions

This paper aims to present a novel numerical simulation method for investigating the seismic behavior of SFRC–BCJs, based on the Opensees analysis software. Meanwhile, parametric studies were carried out. Based on the limited results from the current study, the following conclusions can be drawn:

- A numerical simulation method on investigating the seismic behavior of SFRC–BCJs was proposed by modifying the calculation method of shear deformation in the core area of joint and bond–slip deformation of longitudinal reinforcement of beam. The numerical modeling approach can accurately reflect the development of SFRC–BCJs, and the numerical results agreed well with the experimental results.
- Adding the steel fiber volume ratio can effectively improve the seismic behavior of SFRC–BCJs, in terms of the initial stiffness, yield load, ultimate load, and ductility. Besides, increasing the stirrup amount contribute to enhance the yield load, ultimate load, and ductility. However, the axial compression ratio has no obvious influence on the seismic behavior of SFRC–BCJs.
- Based on the numerical simulation results, the formula for calculating the shear capacity of joints is established. Furthermore, the results show that the proposed formula can reflect the influence of steel fibers and stirrups, which is in good agreement with the numerical simulation results.

## Figures and Tables

**Table 1:** Mechanical properties of reinforcing bars.

Category	*d* (mm)	*f*_y_ (MPa)	*f*_u_ (MPa)	δ (%)	*E*_s_ (MPa)
HRB335	22	418.2	652.1	27	1.95 × 10^5^
HRB335	16	360.5	594.9	23	2.01 × 10^5^
HPB235	8	306.9	472.7	30	2.09 × 10^5^

Note: *d* is diameter; *f*_y_ is yield strength; *f*_u_ is ultimate strength; δ is elongation at fracture; and *E*_s_ is elasticity modulus of reinforcing bars.

**Table 2:** Test parameters of SFRC–BCJs specimen.

Joint Number	Concrete Strength (Mpa)	Volume Ratio of Steel Fiber *V*_f_ (%)	Axial Compression Ratio *n*	Core Area Hoop Reinforcement	Cubic Compressive Strength (MPa)	Split Tensile Strength (MPa)	Elasticity Modulus (MPa)
BCJ1–0	CF60	1.0	0.3	0	81.7	7.3	45,300
BCJ1–1	CF60	1.0	0.2	0	79.1	7.4	43,700
BCJ1–2	CF60	1.0	0.4	0	78.1	7.3	44,400
BCJ2–2	CF80	1.0	0.3	2ϕ8	89.5	7.1	44,500
BCJ3–1	CF60	0.5	0.3	2ϕ8	82.1	7.5	46,600
BCJ3–2	CF60	1.5	0.3	0	86.6	8.9	40,900
BCJ3–3	CF60	2.0	0.3	0	87.4	9.1	44,100
BCJ5–1	C60	0	0.3	5ϕ8	68.6	4.9	42,500

Note: BCJ represents the beam–column joint.

**Table 3:** Mixing proportions of materials per cubic meter.

Number	Water (L)	Cement (kg)	Sand (kg)	Stone (kg)	Steel Fiber (kg)	Superplasticizer (kg)
BCJ1–0	164	547	696	1044	78	8.2
BCJ1–1	164	547	696	1044	78	8.2
BCJ1–2	164	547	696	1044	78	8.2
BCJ2–2	164	547	696	1044	78	8.2
BCJ3–1	156	520	710	1065	39	7.8
BCJ3–2	172	573	682	1023	117	8.6
BCJ3–3	181	599	668	1001	156	8.9
BCJ5–1	146	487	623	1210	0	7.3

**Table 4:** Comparison of feature points.

Component	*P*_y_ (kN)			*P*_m_ (kN)			*P*_u_ (kN)		
	T	S	T/S	T	S	T/S	T	S	T/S
BCJ1–0	27.49	28.55	0.96	35.20	37.65	0.93	32.08	33.41	0.96
BCJ1–1	26.18	24.32	1.08	34.12	37.37	0.91	30.63	33.42	0.92
BCJ1–2	31.64	29.01	1.09	36.45	37.51	0.97	29.88	32.08	0.93
BCJ2–2	32.29	29.99	1.08	39.95	37.66	1.06	33.22	35.10	0.95
BCJ3–1	26.01	26.29	0.99	33.86	34.58	0.98	29.41	30.75	0.96
BCJ3–2	25.74	24.65	1.04	39.27	39.60	0.99	33.87	36.90	0.92
BCJ3–3	27.50	26.38	1.04	40.00	39.00	1.03	33.57	36.02	0.93
BCJ5–1	24.41	23.83	1.02	30.76	30.75	1.00	28.12	30.16	0.93
Average			1.04			0.98			0.94
COV			0.0020			0.0022			0.0002

Notes: T represents test, S represents simulation. COV represents coefficients of variation.

**Table 5:** Comparison between calculated results and experimental results of ultimate shear capacity of SFRC–BCJs.

Joint Number	*V*_jt_ (kN)	*V*_jc_ (kN)	*V*_jt_/*V*_jc_
SF–7 [76]	398.6	393.561	1.013
SF–8 [76]	456.6	486.258	0.939
J3–3 [77]	467.7	454.363	1.029
J3–4 [77]	456.0	503.451	0.906
S3 [78]	1375.5	1238.093	1.111
SF–2 [70]	1087.5	980.296	1.109
S6 [46]	34.1	38.647	0.882
BCJ1–0	348.4	359.264	0.970
BCJ1–1	330.9	344.214	0.961
BCJ1–2	360.5	347.004	1.039
BCJ2–2	384.3	385.718	0.996
BCJ3–1	328.1	404.009	0.812
BCJ3–2	375.9	323.254	1.163
BCJ3–3	390.0	370.185	1.054
BCJ5–1	347.6	360.635	0.964
Average			0.997
COV			0.094

Note: $V_{\mathrm{jt}}$ and $V_{\mathrm{jc}}$ are experimental value and calculated value of ultimate shear capacity in the core area of the joint, respectively.

## Data Availability

Data are available on request from the authors.
